# Protein-Based 3D Biofabrication of Biomaterials

**DOI:** 10.3390/bioengineering8040048

**Published:** 2021-04-16

**Authors:** Mahta Mirzaei, Oseweuba Valentine Okoro, Lei Nie, Denise Freitas Siqueira Petri, Amin Shavandi

**Affiliations:** 1 BioMatter Unit, École polytechnique de Bruxelles, Université Libre de Bruxelles (ULB), 1050 Brussels, Belgium; Mahta.Mirzaei@ulb.be (M.M.); Oseweuba.Okoro@ulb.be (O.V.O.); 2 College of Life Sciences, Xinyang Normal University, Xinyang 464000, China; nielei@xynu.edu.cn; 3 Fundamental Chemistry Department, Institute of Chemistry, University of São Paulo, São Paulo 05508-000, Brazil; dfsp@iq.usp.br

**Keywords:** protein-based hydrogels, 3D printing, peptides

## Abstract

Protein/peptide-based hydrogel biomaterial inks with the ability to incorporate various cells and mimic the extracellular matrix’s function are promising candidates for 3D printing and biomaterials engineering. This is because proteins contain multiple functional groups as reactive sites for enzymatic, chemical modification or physical gelation or cross-linking, which is essential for the filament formation and printing processes in general. The primary mechanism in the protein gelation process is the unfolding of its native structure and its aggregation into a gel network. This network is then stabilized through both noncovalent and covalent cross-link. Diverse proteins and polypeptides can be obtained from humans, animals, or plants or can be synthetically engineered. In this review, we describe the major proteins that have been used for 3D printing, highlight their physicochemical properties in relation to 3D printing and their various tissue engineering application are discussed.

## 1. Introduction

Protein-based materials are abundant, inexpensive, biocompatible, and biodegradable and have been used in numerous applications such as textile, food, cosmetic industry, and biomedical field such as 3D printing of biomaterials. Natural proteins such as collagen, gelatin, keratin, and silk are commonly used as biomaterials. These natural proteins in comparison to synthetic proteins and peptides, have lower immunogenicity, a higher degradability, and enhanced biocompatibility [1,2]. These properties of proteins highlight their possible use as a biomaterial in 3D printing, which may be indicative of the potential benefits of its utilization in the formation of hierarchical tissue constructs [3,4]. The inclusion of proteins in the formulation of biomaterial ink may enhance its tunable mechanical properties, making it comparable to the host tissue and matching its degradation rate with the tissue regeneration [5,6,7]. Proteins inclusion also helps modify biomaterial ink cross-linking properties, modify its viscosity, improve shape integrity for printability and functionalize it for photo cross-linking [5,7,8]. In addition to the stated benefits of protein biopolymer in the biomedical field, using such renewable materials is anticipated to improve environmental performance outcomes from the reduced utilization of fossil-derived synthetic polymers [9,10]. These observations highlight why protein-based biopolymers have been investigated for 3D printing.

Collagen is the most abundant extracellular matrix protein in mammalians which constitutes approximately 35% of the total protein [11]. Structurally, collagen is characterized by a primary sequence of a repeating peptide (Gly-X-Y), where X and Y may be proline or hydroxyproline [12]. The interaction of different peptides leads to the formation of a collagen unit that is based on an altered left-handed polyproline helix. Three of these helix chains can interact to form a right-handed helix structural unit called tropocollagen [13]. Collagen was thought to be nonimmunogenic until the 1950s [14]; however, recently, the immunogenic activity was reported by some researchers [15,16]. Type 1 collagen, the most abundant type, is reported as a poor immunogen compound [17]. Recently, Sapudom J et al. (2020) [18] also reported that type I collagen does not trigger an inflammatory response and macrophage activation, suggesting the collagen’s biocompatibility. Pepsin-treated collagen (atelocollagen) has been widely used in regenerative medicine and commercial products due to its immune compatibility [19]. The telopeptide domains with the highest flexibility are found as the most immunogenic domains of collagen. By removing these regions enzymatically, it is possible to reduce the immunogenicity. However, this is not possible for tissue-based biomaterials since they are the site of crosslinking. However, on the other hand, the immunogenicity can be partly reduced through crosslinking with glutaraldehyde [17].

Gelatin, as the denatured collagen protein, is another common protein for 3D printing that has a similar composition to collagen. Gelatin cannot normally be found in nature and is obtained by partially hydrolyzing collagen, under the action of an enzyme such as neutrase [20] or under the action of an acid or an alkaline [21]. Keratin, abundant in feather, wool and hair, is another protein that can be classified into two different categories: alpha-types and beta-types [1]. These protein types may dissociate in terms of their pattern of filament formation [1]. The alpha-type keratin has a helical structure with a diameter of 7 nm, while the beta-type keratin has a beta-sheet type of structure with a diameter of 3 nm. Keratin contains a high number of cysteine residues that are responsible for the many intramolecular and intermolecular interactions, leading to higher stability and lower solubility of the protein [1,22]. Keratins are usually insoluble because of the disulfide bonds [23,24].

**Figure 1:**
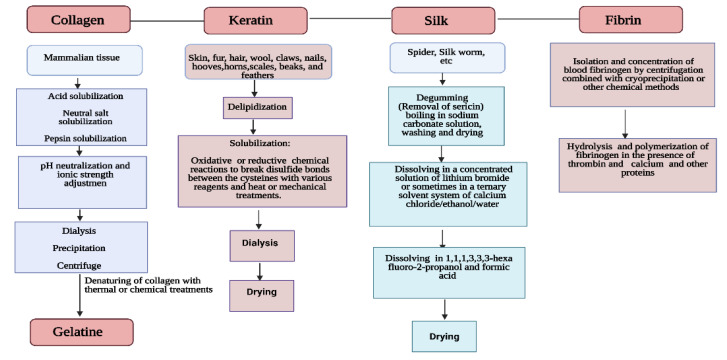
Sources, production and purification methods of some of the protein-based biomaterials.

Further studies showed that α and β-type keratins exhibit high content of half-cystine, glutamic acid, glycine, proline, and serine amino acids [25]. Regarding the immunogenicity activity, Funjii et al. [26] examined the antibody-producing activity against different keratin from hair and nails and reported no rejection or allergic reactions in mast cells. The silk fiber is spun by several arthropod species and may be used to produce luxury textiles with exceptional texture and robustness [27]. Other unique properties of silk such as high transparency, mechanical robustness, and the possibility to be patterned at multiple length scales, have led to the development of several bio-optical devices [27]. Silk is made of fibroin, a protein consisting of a succession of G, A and S amino acids [28]. Given the arrangement of these proteins, the structure will be type I, II or even type III. While type I silk originates from silkworms and exhibits metastable crystalline structure, type II refers to an arrangement where β-sheet structure is dominant [29]. Type III was discovered at air–water interface and leads to a hexagonal packing of silk filaments in a left-handed threefold helical crystal structure [30]. Silk fibroin is popular in the biomedical field due to its advantageous properties, including high biocompatibility, diminished immune reactivity and good mechanical properties. It has been demonstrated that silk scaffolds exhibit low cytotoxicity and can promote angiogenesis in vivo [31]. The silk scaffolds with high porosity, designed for tissue engineering, allow blood vessel growth. The growth can be induced by preseeding of osteoblasts and endothelial cells before implantation or by adding biological signaling molecules or through structural modification [31]. Fibrin is a nanofibrous protein involved in blood clotting and wound healing; it is formed by the enzymatic cross-linking reaction between thrombin and fibrinogen [32]. In its cross-linked form, fibrin hinders proper extrusion during printing [33]. Therefore, it is available as fibrinogen and needs to be combined with a cross-linking solution of thrombin and an ionic binding agent. Printing is possible using a support bath containing the cross-linking solution or by in situ cross-linking where the biomaterial ink and cross-linking solution are individually extruded from separate needles, solidifying at the end of the nozzle [33]. After cross-linking, the fibrin forms a complex and stable network that allows a high degree of deformation. Fibrin is applied in the fabrication of skin grafts and to recreate the natural wound-healing environment found in skin tissue [32]. The silk scaffolds exhibit less cytotoxicity and can promote angiogenesis in vivo [31]. The silk scaffolds with high porosity, designed for tissue engineering allow blood vessel growth and the growth can be induced by preseeding of osteoblasts and endothelial cells before implantation [34] or by adding biological signaling molecules or through structural modification [35]. The advantages and disadvantages of some protein-based materials with a special interest in their applicability in tissue engineering are summarized in Table 1 and a summary of the production and purification methods of each one is presented in Figure 1.

## 2. Protein-Based Ink Exploration

### 2.1. Biofabrication Techniques Used for Protein-Based Ink

It is well-known that selecting the appropriate biofabrication technique for different biomaterial inks is one of the most crucial and challenging steps to obtain high-efficiency rates of the production process. Choosing the right biofabrication method for a certain ink is mainly determined by the purpose of the application. In this target tissue, the application will be performed and the biomaterial ink type [45]. Moreover, chemical, biological, and mechanical properties of the ink must be identified since the advantages and disadvantages of each biofabrication technique will differ depending on these features. A literature review shows that the major printing techniques include inkjet printing, extrusion printing, laser-based printing, stereolithography, electrospinning, and melt electrospinning. The major advantages and limitations of these biofabrication techniques are summarized in Table 2.

Table 2 shows that despite offering high resolution, inkjet printing solely allows the use of low-viscosity ink formulations, thus may not be applicable for use with proteins. Table 2 also highlights that extrusion printing is particularly useful since it offers the advantage of printing biomaterials within a wider viscosity range. Laser-based printing is amongst the options for biofabrication purposes since it offers high resolutions, and it is suitable for a wide range of biomaterial ink viscosities. Considering all the methods highlighted in Table 2, inkjet printing and extrusion printing techniques can be identified as the most preferable for proteins such as silk. This is because silk-based inks can be tuned and adjusted by adding other biomolecules such as enzymes, growth factors, nanoparticles, and their properties can be modified by changing the silk concentration or molecular weight. For example, by adjusting the molecular weight, ink surface tensions between 0.04 and 0.07 N/m and dynamic viscosities between 3 and 300 mPa s were obtained [36]. Moreover, silk-based hybrid inks (e.g., silk-hydroxyapatite) present improved characteristics when printed. Another printing technique that may be employed when using (some) proteins is stereolithography. This method is known for its high resolution and accuracy due to the light patterns used. Yet, there is not a great number of materials suitable for the use of stereolithography, which would also be limiting for protein-based inks. Even though collagen is a protein-based ink that is capable of being cross-linked by ultraviolet lights, this cross-linking does not take place fast enough for printing to be successful unless collagen molecules are functionalized. On the other hand, as another example of protein-based ink, gelatin displays similar properties to collagen, and it can be more easily photopolymerized than collagen. For instance, gelatin methacrylate is currently gaining a lot of interest in tissue engineering due to its cross-linking properties [46].

Additionally, electrospinning has been employed in fabricating protein-based inks. This is because proteins such as collagen, fibrinogen and gelatin were suitable for electrospinning in terms of structural properties. However, the positioning of these fibers may interfere with pore sizes and the volatile solvents used might be toxic and can deteriorate the protein-based ink. Solvents have been designed to avoid such issues. Still, another solution to this problem can be melt electrospinning, where the solidification mechanism depends on cooling instead of solvent evaporation without the need to use a solvent [47]. Yet, this method is limited in terms of viscosity, unlike electrospinning, which is why it would need to be further tested on viscous protein-based inks like collagen, depending on the ink formulation. To employ protein-based materials in biofabrication, it may be necessary to explore cross-linking techniques. The cross-linking of protein-based materials is discussed in the subsequent section.

### 2.2. Cross-Linking of Protein-Based Materials

#### 2.2.1. Mechanism of Cross-Linking

The cross-linking methods may differ in several aspects, such as reactivity and functionality. Cross-linkers are categorized by their ability to target the functional groups of proteins as monofunctional cross-linker or multifunctional cross-linker. Thus, the monofunctional cross-linker works with one functional group and bifunctional or multifunctional cross-linker work simultaneously with two or more functional groups. By studying the reversibility of the cross-links, it is possible to distinguish dynamic cross-linking with static. A static network is composed of covalently nonreversible cross-links. In contrast, dynamic linkages have reversible cross-links. It allows the polymer the ability to repair internal damage and recover its original shape. For protein materials, self-assembly of the alpha-helix and beta-sheet is impacted by the hydrogen bonding. The study by Saiani et al. [49] reports that strong hydrogen bonding interactions along the fiber axes linking each peptide with the previous and the next one.

The consequence is the creation of a very stable structure. This system is not very sensitive to the polar group position. In comparison, the interpeptide interactions of an alpha helix are weaker. In this system, the position of a polar group plays a key role in the self-assembly mechanism [49]. Crosslinking can be performed by enzymes (e.g., Transglutaminas and Lysyloxidase) or with various natural (glucose, ribose, riboflavin, genipin) or chemical (glutaraldehyde, carbodiimide(1-ethyl-3-(3-dimethyl aminopropyl)-carbodimide (EDC) crosslinkers. Enzymes used as catalysts to promote the formation of covalent bonds and the cross-linking reaction using enzymes may be controlled by changing temperature or pH. The enzymes used for crosslinking are not toxic and can be used in presence of cells.

Enzymes are also biocompatible and can react at body temperature in a physiological environment. These factors explain the interest of this method for synthesis of injectable in-situ protein-based hydrogels. For example, transglutaminase enzyme is a good candidate for cross-linking in various fields such as protein hydrogen, food protein, or protein fibers and leather [50]. Natural crosslinkers are also less toxic than chemical ones. They have been used to increase matrix stiffness without any toxic effects on cells. For chemical crosslinker, the toxicity and calcification are the problems reported for glutaraldehyde. Although, EDC is not toxic and is known as a zero-length crosslinker that forms amide bonds between carboxyl and primary amines [51,52]. McKegney et al. [53] considered the effect of crosslinking treatments of collagen with EDC, diamine and diaminohexane on pore size, morphology and stability of crosslinked sponge. They reported unlike glutaraldehyde, these chemical agents were not toxic against fibroblasts and keratinocytes. Crosslinking reduced the pore size, especially at the surface and altered sponge morphology. Collagen fibers became thinner and influenced on sponge stability [53].

Notably, an interesting gelation type is ionotropic gelation, which enhances the self-healing of a material. The ionotropic gelation method is a physical cross-linking method based on electrostatic interaction and is a simple and low-cost process. Moreover, physical cross-linking avoids toxic reagents or unwanted side effects compared to chemical cross-linking. Cross-linking constitutes an important aspect being considered when handling protein-based material; for instance, for cross-linking of insulin to chitosan (CS) nanoparticles [54], CS was dissolved in aqueous acid and the NH groups of the CS molecule were activated to obtain a CS cation. The resultant solution was mixed with tripolyphosphate (TPP) in a constantly stirring environment for enhanced mass transfer interactions. The TPP acts as a polyanion with the negatively charged phosphoric anions reacting with positively charged cationic CS to form cross-linked chitosan nanoparticles [54]. Additionally, physical irradiation methods have been used to improve the thermal stability and swelling properties with maintaining chemical structure without the introduction of any cytotoxic reactant [51]. In contrast, physical crosslinking using electron beams reduces the porosity of collagen scaffolds [55].

The UV method enables free radicals on aromatic groups, which will form chemical bonds with each other. However, using only UV, high cross-link density in protein materials cannot be produced [56]. Photo cross-linking is a method with good control of gelation timing and kinetic due to ultraviolet light initiation. Ionic cross-linking with multivalent cation, often calcium ion is usually used in this method. It is, however, noted that the application of the UV method for cross-linking purposes could damage cells. Furthermore, some photoinitiators are cytotoxic in precursor or radical form. The use of the UV method in cross-linking of protein-based materials can also lead to the generation of bubbles or temperature differences that affect the cross-linking [57].

#### 2.2.2. Cross-Linking of Protein-Based Materials

Protein-based materials can be cross-linked via physical, chemical, and enzymatic methods. For instance, in the chemically induced cross-linking of collagen molecules, glutaraldehyde can be used. However, the exact mechanism of interaction between the collagen and this molecule is not completely clear. It is, however, hypothesized that the amino group of lysine would react with the aldehyde group of the glutaraldehyde. This cross-linking allows stabilizing collagen against thermal degradation [56]. The gelatin, on the other hand, can be cross-linked via physical gelation, which is a reversible and unstable technique of cross-linking, as discussed in Section 2.2.1. This is because in solution and at high temperature (40–50 °C), gelatin shows a specific structure of reverse random coil, which during cooling shows a transition thanks to hydrogel bonds stabilization into a triple helix. Tyrosinase and transglutaminase can be used to produce a stable and biocompatible cross-linked network of gelatin [58].

As reported by Rutz et al. [8], gelatin can be further functionalized to offer finely tunable cross-linking properties. Functionalization was reported to be achievable with thiols and tetrazines via amine-reactive molecules, 2-iminothiolane (Traut’s reagent) 1,2,4,5-tetrazine-C5-N-hydroxysuccinimide, respectively [42]. These cross-linking properties were reported to offer moduli ranging from 500 Pa to 2 kPa, which are suitable for most soft tissue and achieve strain promoted click chemistries of a matrix showing tetrazine functional groups with norbornene and activated amine groups with esters, thus, allowing the building of constructs with spatially and temporally complex mechanical and biochemical microfeatures. Furthermore, it was reported that with post cross-linking, the viability of 80–90% of cultured cells was maintained, thus proving to be viable when applied as ink. Additionally, keratin can be cross-linked by adding a photosensitive compound (for example, riboflavin-SPS sodium persulfate-hydroquinone) in the solution and then using UV to induce the formation of a link between the monomers [42]. Keratin can be incorporated into a membrane using a guided tissue regeneration-based membrane. This modification alters the surface properties of the membrane leading to an increase in its roughness and hydrophilicity that could enhance cellular behavior. This modified membrane can be applied in the medical field for soft tissue regeneration [59].

## 3. Proteins-Based Materials and 3D Printing

### 3.1. Status of Protein-Based Inks for 3D Printing 

State of the art in printing tissue modeling is constantly improving. However, tissue engineering lacks the ability to reproduce optimal biomimetics and replicate the tissue heterogeneity because of the absence of appropriate biomaterials and technologies [60]. To tackle this issue, using multiple building blocks, sacrificial biomaterials, and cell types in a single ink for artificially printed tissues offers a promising solution to reproduce the heterogeneous character of tissue composition, thus ensuring a fully functional artificial tissue. The protein-based material of collagen is rarely used alone due to its poor mechanical properties and fast biodegradation rate [61]. Thus, it requires mixing with other polymers to improve its mechanical properties while retaining the biocompatibility of the overall material and cell proliferation [61]. The proportions of collagen (protein) used will also influence the material’s transition temperature and lead to a decrease in the gel transition temperature. For instance, Peng et al. used collagen to modify the properties of chitosan/β-glycerophosphate based biomaterials with LCST behavior near body temperature. This behavior is used in the delivery of venlafaxine hydrochloride [62]. Notably, the compressive strengths of cross-linked silk fibroin (SF) and glycidyl methacrylate to produce SF methacrylate hydrogels, were similar to the compressive strength of typically modeled tissues such as 3D-printed artificial mitral valve leaflets (<100 kPa), smooth muscle (6–10 kPa), and carotid artery (84 kPa) [38]. The degradation rate of the formed scaffold was also similar to tissue regeneration for bone cartilage tissue [38]. Another study showed that increasing the ink fibroin composition made the scaffold stiffer and further slowed down the degradation rate [63]. Ghosh, et al. [64] developed a photo cross-linkable SF methacrylate ink to print tubular and solid organ models, mimicking the real tissue. Such models mimicked the heart, lungs, trachea, vasculature, ear, and brain and showed good cell viability [7,38]. Jiang, et al. [65] also reported the fabrication of a particular 3D printing involving an assembly of two biomaterials of collagen and silk fibroin to treat spinal cord injury.

To overcome the gelatin’s poor mechanical properties, the gelatin hydrogel may be modified using silk fibroin [37]. For instance, combining gelatin with SF and sulfonated SF, facilitated enhanced mechanical properties without compromising the biocompatibility and nontoxicity requirements [37]. Recently, SF has also been employed in mesoporous bioactive glass (MBG)/SF composites to fabric scaffolds by 3D printing [66]. The MBG/SF scaffolds were shown to present superior compressive strengths (ca. 20 MPa) and good biocompatibility. The stimulated bone formation ability was improved compared to more commonly employed mesoporous bioactive glass/polycaprolactone (MBG/PCL) scaffolds. Thus, SF may be considered a promising candidate for bone tissue engineering. Furthermore, the potential of a novel blend of silk scaffold has also been investigated by blending mulberry (Bombyx mori) silk fibroin with a non-mulberry (*Antheraea assamensis*) silk fibroin which is rich in a cell adhesion protein (arginine-glycine-aspartic acid) to produce a functional liver construct [67]. The results showed that blending mulberry and non-mulberry silk fibroin would help to form stable and optimally sized hepatocyte clusters (<100 µm) with enhanced functionality which might enable better nutrient and oxygen diffusion. Notably, some protein-based materials employed in inks have unique properties such as being stimuli-responsive and having self-assembling characteristics.

### 3.2. Protein-Based Stimuli-Responsive and Self-Assembly Inks

Stimuli-responsive protein-based inks are an important emerging class of inks that can respond to a specific external stimulus and can alter their physicochemical properties (i.e., functionalities, shape, hydrophobic/hydrophilic behavior etc.) and rheological properties when subjected to an external stimulus in the living body or during the fabrication process [62,64,68,69]. Indeed, these external stimuli can originate from different sources including but not limited to pH variations [64,69], thermal variations [62,68], photosensitivity [68], and ion concentrations [64]. The ability of this new class of biomaterials and the produced scaffolds provides new possibilities for tissue engineering. In this section, we will highlight different applications of thermo-responsive protein-based materials and pH-protein-based responsive materials in 3D printing [62,64,68,69]. It is important to highlight certain points about protein-based biomaterials, which are their relative ease of production, safety, their similarity to the extracellular matrix, and their biocompatibility [62]. The application of stimuli-responsive proteins was demonstrated in the work of Ghosh, Barman, Sarkar and Ghosh [64] who showed that peptide-based hydrogels responded to stimuli by exhibiting a gel state at neutral or weakly basic pH (pH = 7.4) and a non-gel state when the surrounding solution is acidic. Thus, these peptide-based hydrogels could release chemical agents as the pH becomes acidic.

The self-assembly characteristic of protein-based materials is present in nature. All organisms are formed through self-assembly; for instance, from the zipping of two DNA strands into a double helix, the encoding human genome to cells gathering and forming tissues, organs, and complete human bodies was achieved via self-assembly. Integrating self-assembly into printing allows creating structures with high levels of hierarchy, complexity, and functionality by aggregating molecules with noncovalent interactions. The self-assembling property also allows eliminating UV/visible light exposure to achieve photopolymerization. Peptide and protein-based self-assembling inks offer an approach to recreate, for example, extracellular matrix (ECM) elements. Self-assembling inks gained interest in the printing field as they can reorganize into structures with enhanced properties that cannot be achieved with current inks and printing methods. For example, SF is known to undergo gelation at room temperature by the self-assembly of its beta-sheets [70]. SF is widely used in biomedical applications and tissue engineering. When added to gelatin, the formed hydrogel’s mechanical properties can be tuned depending on the load of silk fibroin [71]. Kulkarni, Guha Ray, Byram, Kaushal, Dhara and Das [71] investigated the physico-mechanical, chemical, and biological properties of the tailored matrix formed by the silk and gelatin. They achieved dual cross-linking of gelatin and activation of fibroin and maximal cell adhesion and growth by adding silk fibroin. The improvement of mechanical resilience and cytocompatibility of the gelatin led to the formation of a superior hydrogel than gelatin silk-free based hydrogel. Ultrashort peptides have previously also been used to undergo gelation under physiological conditions to self-assemble in stable, nanofibrous three-dimensional hydrogel scaffolds [72]. The peptides, driven by an amphiphilic motif, assemble in an antiparallel fashion from random coil to alpha-helical intermediates and beta-helical fibrous structures that finally condense into three-dimensional networks [72]. The amphiphilic characteristic of the peptides can trap water, forming hydrogels, and avoiding dehydration during printing. These scaffolds could support stem cells’ cultures while staying stable and their inherent in vivo biocompatibility makes them advantageous for regenerative medicine applications. Hedegaard et al., [63] worked on a hydrodynamically guided biofabrication system with peptide amphiphiles that coassemble with keratin to form a nanofibrous hydrogel that could be chemically and mechanically tuned by changing the component ratio. The hydrogel was printed by droplet jetting and showed promising and stable results during its incubation time. The droplet-on-demand inkjet printing was completed using interfacial forces generated during the process between coassembling molecules resulting in a guided self-assemblage into complex geometries. The same study investigated collagen, the predominant protein in the ECM, based ink to assess their system’s versatility and subsequently confirmed its potential for printing [63].

### 3.3. Nanoparticle or Nanofiber Reinforced Protein-Based Inks

There is a real need in tissue engineering for inks that are both mechanically stable and biocompatible [73] and would enable the additive manufacturing of structures with increased precision, an increased complexity, a greater aspect ratio, and a reduced maturation time [73]. In this regard, a promising area of research lies in nanocomposite inks containing nano-biomaterials. These nano-biomaterials can originate from both organic and inorganic sources and are generally classified into four major categories of namely, ceramic, polymer, carbon, and other biomaterials [74]. Within these four categories, the nano-biomaterials can be found in multiple forms ranging from tubes, fibers or simply nanoparticles. This section will look at recent progress that has been made in this field of research using different categories of nano- protein-based materials as examples.

In the realm of polymer biomaterials, a division can be made between natural and synthetic polymers. In their work, Clark, Aleman, Mutkus and Skardal [73] focused on formulating a mechanically stable collagen and hyaluronic acid (HA) ink by incorporating gelatin nanoparticles (GNP). These nanoparticles were produced in the form of a powder and mixed with a collagen solution to hydrate and disperse the GNP in the hydrogel. Rheological characterization showed that the ink was robust, self-supporting, and thixotropic. The addition of 150 mg/mL of GNP to the ink was enough to increase the storage modulus from 2 Pa to 3.3 kPa. A thixotropic ink indicates that it demonstrates shear thinning behavior and could act as a solid under low shear conditions and as a liquid when a critical shear strain is attained, which is ideal for extrusion printing. Complex models of intersection vessels were printed without support and remained free-standing and proliferation studies also indicated that this ink was suitable for cell growth [73]. Carbon nanotubes (CN) are known for their unique properties and have been used extensively in composite materials. Zhu, et al. [75] investigated the possibility of adding carbon nanotubes in a gelatin-alginate ink formulation to produce artificial vessel constructs. The goal was to produce composite hydrogel constructs that could withstand similar strong mechanical forces that blood vessels experience under physiological conditions. The constructs produced with 0.5% CN showed Young’s modulus of almost five times higher than the control structure and good cell proliferation with a cell adhesion rate of around 80%.

On the other hand, when 1% CN was added, a critical concentration was reached, and cytotoxicity was observed which is in part due to the poor cell affinity of CNs [75]. Proper dosing of CNs in the ink is important to obtain a good balance between improved mechanical properties and cytotoxicity. A future area of research could focus on surface modifications of CNs to improve their poor cell affinity and on the effects of their high conductivity on cells [75]. These examples are just some of the numerous studies ongoing in the field of nano-biomaterials and multiple composite materials containing, for example, nano-silicates [76] and silk fibroin are currently being investigated. These reinforced structures can pave the way to more possibilities in the field of tissue engineering, for both soft and hard tissues, using constructs with improved mechanical performances, enhanced biocompatibility, and tailored properties to fit any application throughout the human body. Nanocomposites have shown how useful and promising they are in numerous other scientific fields and will more than shape the future of tissue engineering. More research needs to be done in these nanocomposite biomaterials as the field attempts to edge closer to clinical applications. Table 3 summarizes some protein-based biomaterials employed in 3D printing as reported in the literature.

## 4. Biomedical Application of Protein-Based 3D Printed Materials

### 4.1. Skin

Skin tissue engineering is an important part of 3D printing applications and biomedicine in general. The skin is the largest organ of the human body, and therefore, it is easily damaged in accidents [84]. The use of gelatin-sulfonated silk composite scaffold was proposed by Xiong, Zhang, Lu, Wu, Wang, Sun, Heng, Bunpetch, Zhang and Ouyang [37] in skin treatment. This scaffold was 3D printed and incorporated with basic fibroblast growth factor 2 (FGF-2) through binding with a sulfonic acid group (3DG-SF-SO_3_-FGF), to enhance the treatment efficacy. Incorporating bioactive compounds in the 3D scaffold as reported by Ramanathan, et al. [85] is another example of fabrication of a 3D collagen (COL-SPG), which was replicated and impregnated with an antibacterial drug (COL-SPG-D) and a bioactive CPE extract (COL-SPG-CPE). The results suggested the 3D COL-SPG-CPE spongy scaffold could serve as a potential wound dressing material.

Delivery of cells to the wound using techniques such as cell spraying or manually seeded matrix results in faster healing of wounds for improved cosmetic outcomes compared to wounds healed using noncellular substitutes [84]. However, these techniques are not able to obtain the desired result due to their low delivery precision. In this context, Albanna et al. [84] reported a technique based on layer-by-layer in-situ printing to deliver dermal fibroblast and epidermal keratinocytes to specific locations of the wound. Firstly, this technique was investigated via an in vivo test in a mice group and demonstrated faster wound closure (3 weeks) compared to untreated and matrix-treated groups (5 weeks). Furthermore, the bioprinter was evaluated in a porcine wound model, where allogeneic and autologous fibroblasts and keratinocytes were tested. The results suggested that the in situ printing of autologous cells resulted in a 3-week acceleration in the wound closure compared to allogeneic cells. The analyses showed epithelialization by week four in the case of autologous cells, while allogeneic cell and matrix-treated wounds did not show epithelialization until week 6. Untreated wound healing appeared even more delayed. Autologous in situ printing treated wounds showed accelerated wound closure, reducing wound contraction, and increased re-epithelialization.

### 4.2. Bone

Bone tissue engineering involves creating a cell-seeded scaffold using an in vitro culture of bone tissues on an artificially built scaffold. The cell-seeded scaffold is implanted into the defect/damaged site to cause cell multiplication for bone recovery [86]. Within human tissues or organs, bones are considered as a stiff material, and, therefore, hydrogels used in bone tissue engineering must have a high stiffness to mimic these hard tissues and should also exhibit a non-Newtonian behavior to be effectively printed [87]. The two major constituents of bone are hydroxyapatite and collagen, which both are usually used in bone reconstruction. Collagen-hydroxyapatite (CHA) scaffolds benefit from combining the mechanical strength of ceramics with the biological advantages of collagen [88]. Usually, collagen-containing scaffolds with a high level of cross-linking show a higher printability and porosity with proper diffusion of the nutrients for cell activity [89,90].

One way to achieve the regeneration of large bone defects is by reproducing the whole bone architecture to the fabrication of a 3D large-scale bone tissue with functional vasculature, as proposed by Byambaa, et al. [91]. To successfully fabricate the 3D structure, the extrusion-based printing method was used to construct microstructured bone-like tissues. The bioprinted constructs were used as biomimetic in vitro matrices in a naturally derived hydrogel to coculture human umbilical vein endothelial cells.

### 4.3. Cardiovascular Tissue

Cardiovascular disease (CVD) is one of the leading causes of death worldwide, with high mortality rates reported in older people [92]. The high mortality of CVD is partial because ‘self-healing’ of the tissues in the cardiovascular system is difficult to achieve with the replacement of tissues like heart valves or myocardium, usually determined to be the most effective treatment [93]. Nowadays, the replacement methods are mainly autografts and allografts. Still, the side effects during implementations like donor tissue shortage, immune rejection and inflammations become the main drawbacks that are hard to ignore [93]. Recently, an emerging new technique, 3D printing of cardiovascular tissues, has provided an alternative strategy for treating CVD via its use in the fabrication of vascular constructs. A notable alternative strategy is called sacrificial printing. As its name implies, in sacrificial printing [94], the vascular network is filled with sacrificial ink, which can be removed by temperature changes or specific solvents and surrounded and supported by relatively rigid matrixes. Depending on both the nozzle diameter and printability of ink, a multiscale vascular network could be fabricated. According to a recent study by [95], thermosensitive protein-based materials such as gelatin hydrogels can be used as sacrificial inks for their good biocompatibility and biodegradability. The second method is called coaxial nozzle-assisted printing. It is mainly based on using a coaxial nozzle to create filaments with a hollow structure which could act as a structure in the scaffolds for the delivery of nutrients. In this process, the biomaterial is extruded out through the outer tube of the coaxial nozzle and cross-linked after it contacts with calcium chloride, then the gelled alginate becomes the “rigid wall” of the channel during the formation of the vascular structure. This technique’s advantage is the precise control of the geometry, length, orientation, and diameter. The major disadvantage of this strategy is that the ink needs to have a fast cross-link ability, which limits the choice for the ink [96]. The regeneration of cardiac tissue using 3D printing for scaffolds fabrication has previously been demonstrated using protein-based materials of gelatin [95] and collagen [97,98].

### 4.4. Liver Tissue

Protein-based materials have been employed in the fabrication of 3D liver tissue. For instance, in the work of Yang, et al. [99], the construction of a 3D hepatorganoids mouse liver model has been reported both in vitro and in vivo. The bioprinted hepatorganoids (3DP-HOs) were printed using an extrusion printer and fabricated using hepatic stem cell line (HepaRG) and employing the protein-based alginate/gelatin as ink. After seven days of in vitro differentiation, liver functions of the 3DP-HOs were observed and then were transplanted into the abdominal cavities of these mice with liver failure. In terms of ink and printing technique, the group reported that alginate/gelatin was a perfect ink candidate since the ink was biocompatible and resulted in a good structure. These findings are consistent with the work of Hiller, et al. [100].

Moreover, the study indicated that cell survival rate and 3D durability are affected by the composition of ink and printing conditions such as nozzle temperature. In terms of liver functions in vitro, the authors noted that after 2–3 weeks of culturing, an increase in liver functions of 3DP-HOs was detected. After transplantation of 3DP-HOs into mice with liver damage, mice survival time was significantly increased with a decrease in body weight loss. Thus, the use of protein-based materials in liver tissue applications is worthy of further in vivo investigations to establish viability.

### 4.5. Nervous System

The central nervous system (CNS) is subjected to many forms of damage, such as many neurodegenerative diseases (i.e., Alzheimer’s disease, Parkinson’s disease, etc.), injuries due to accidents and tumors such as glioblastomas [101,102,103]. For this reason, 3D printing has been attracting much attention in the field of regenerative medicine for the CNS since 3D printing can be used to construct in vitro models to simulate the disease propagation in the human brain, therefore allowing research groups and drug companies to test their drugs in a working system [101,102,103]. The use of 3D printing is considered better than 2D cell culture models due to the possibility of the constructs to mimic the nervous system microenvironment along with cell–cell and cell–matrix interactions, present in the central nervous system [101,102,103]. Moreover, 3D printing could be used to effectively regenerate the peripherical nervous system (PNS), [104] and nerve guidance conduits (NGCs), inhibiting scar formation and preventing compression of regenerative nerves as well as providing a bridge to fill longer gaps [104,105].

The protein-based material of collagen constitutes a major component of the peripheral nerves ECM. It exists in the layers of the perineurium and endoneurium as fibrils with type III and V collagen. It can be easily transformed into tubular and fibrillar structures thanks to its high extrudability [106]; Schwann cells can also adhere to it, promoting the formation of myelin [107]. Since the protein-based material of gelatin dissolves at normal human body temperature, the GelMA (gelatin modified by methacrylic acid) scaffold is considered more useful in neural tissue engineering applications [107]. The primary carboxyl groups of gelatin can bind amine groups of bioactive molecules, thus allowing the attachment of neurotrophic factors (NTF) which can then be gradually released during gelatin degradation [106]. Silk fibroin is also a useful biomaterial for creating nerve conduits due to its favorable properties of resistance to breakage and compression (high resilience) [106].

Nevertheless, many problems arise from the use of the protein of silk in the field of neural tissue engineering, such as insufficient mechanical support and cytocompatibility, which may be addressed by using multihydrogel mixtures or composites [105]. For instance in the work of Jansen et al. [101], matrigel protein was used in combination with other materials to create a scaffold laden with cortical neuron cells taken from mice. The results showed the possibility to rapidly create a functional model and it broadened the range of possibilities to study neural functioning in a 3D model in both normal and disease situations. In the field of PNS regeneration, Ye et al. [104] investigated the possibility of regenerating peripheral nerve through GelMA-based multichannel nerve guidance conduits (NCGs), showing that this procedure was indeed feasible and could lead to better and faster axon regeneration. Another fundamental property of neural tissues in both CNS and PNS injury treatment, but which is not always addressed by research groups, is conductivity. Conductivity is another driving force for neuron proliferation and differentiation, it could be implemented in hydrogel-based scaffold by means of metal nanoparticles (NPs), carbon-based materials and conductive polymers [108]. The major advantages of metal NPs are their conductivity, magnetic and antibacterial properties as well as the relative ease of tuning of these properties by playing on shape and size. About carbon-based materials, their major advantages are the excellent biocompatibility, wide range of physical and chemical properties, high surface area and conductivity. Nevertheless, both these options show long-term unsolved cytotoxicity, thus preventing their use in neural tissue regeneration. Vijayavenkataraman et al. [108], therefore, studied the possibility of fabricating a conductive collagen-based hydrogel scaffold combined with a polypirrole-b-polycaprolactone (PPy-b-PCL) biodegradable and conductive copolymer. The study focused on rheological properties of the scaffold and showed that indeed it was possible to use this combination, for enhanced conductivity.

Notably, several studies exist in the literature that has explored the use of protein-based materials in brain tissue applications, highlighting the importance of the brain in the CNS. The various studies that have employed protein-based 3D bioprinted materials in brain tissue applications are summarized in Table 4.

## 5. Commercially Available Protein-Based Inks

Biomaterials can be synthesized in a regular lab. There is, however, a batch-to-batch difference in the composition and mechanical properties, creating margins of inconsistency. Purchasing a commercial biomaterial ink ensures reproducible results [110]. In addition, by using commercially available standardized biomaterials, scientists would study organ and tissue models on a larger scale with a unique ink formulation. On this basis, different companies produced different kinds of biomaterials with specific applications. Thus, there is a market for advanced biomaterials developed specifically for 3D printing technology. Table 5 provides a list of some of these commercially available products. Among these products in Table 5, gelatin methacrylate (GelMA) produced by Gelomics company constitutes an example of the gelatin-based material and is produced from the conjugation of methacrylate groups to the amine side groups of gelatin. This biomaterial presents favorable properties such as biocompatibility, enzymatic cleavage and adjustable mechanical properties and can be used in versatile applications such as tissue engineering, drug delivery, and 3D printing. It is also available in blends with nanofibrillar cellulose, alginate or xanthan gums which combine the advantages of both components and ensures smooth printability. It can also be modified with other proteins such as laminins and fibrinogen to create favorable environments for all cell types. BioGelX produced different types of synthetic peptides and modified synthetic peptides hydrogel inks with gelation achieved independently of variations in pH and temperature or contain peptides as surface ligands for enhanced functionality. There are also biomaterial inks available that exist as a mixture of different ECM proteins, as shown in Table 5. For instance, in Table 5, Corning® Matrigel® is a gelatinous mixture that is composed of type IV collagen, laminin, and entactin/nidogen and also proteoglycans and growth factors. It is derived from Engelbreth-Holm-Swarm mouse sarcoma cells, which makes it not appropriate for clinical applications, but it can be used to print cancer spheroids and is vital in the discovery of novel cancer treatments.

## 6. Factors Hindering the Applicability of Protein-Based Materials in 3D Printing

When designing an ink, two aspects have to be considered: the physical requirements and the biological requirements. The physical properties of ink consist of multiple parameters like printability [118], mechanical integrity [119], degradation behavior, the ability to be functionalized [118], and a structure suitable for mass transfer [119]. For ink to have good printability, the ink should have good processability and good print fidelity. These properties are linked to the viscosity of the solution, its surface tension, its gelation process and possibly its shear-thinning characteristics [118,120]. To create a ‘self-standing’ structure, sacrificial support materials can be used [120], or hybrid inks can be created [119]. A drawback of these hybrid inks is that they are more complex and have less control over the cell’s responses [121]. Collagen, for instance, has low mechanical properties. This problem can be remediated using supportive gels, such as a gelatin slurry that can act as a thermo-reversible support. However, this approach presents its own limitations as the gelatin from the support gel can diffuse into the ink [97,122,123,124]. Another way to improve the mechanical properties of collagen is to add different polymers in various proportions but this method can alter the biocompatibility of the hydrogel [97,122]. As for gelatin, it is very rarely printed in its native form due to the poor mechanical properties. Gelatin undergoes chemical cross-linking by the addition of agents such as glutaraldehyde to be employable in printing. [123].

Another important aspect is the biological requirement of the biomaterial. Nontoxicity, degradability, cell adhesion property, biocompatibility, nonimmunogenicity, and porosity are important factors determining the compatibility of biomaterial ink with living organisms. The biomaterial should not be toxic, and fabrication methods should not induce the production of toxic compounds. Laser-based bioprinting may damage the cells with heat and shear stress produced during extrusion bioprinting can also be harmful to cells. The density and viscosity of bioinks are important factors in cell viability because cells need a porous environment for growth. Additionally, the low viscosity of bioink can reduce the shear stress during the extrusion process [125,126,127].

Furthermore, proteins in protein-based inks usually need to be functionalized with an appropriate cross-linking mechanism. Photo-cross-linking inks are often used because the process is fast and provides good control. However, photo-initiators and UV-light can be cytotoxic, thus preventing this technique from being used in situ [128,129]. Chemical cross-linking, however, will most of the time result in some sort of cytotoxicity. For glutaraldehyde (GA) this is the case since it can cross-link protein, which can cause dysfunction in cells. Carbodiimide hydrochloride (EDC) can be used as a chemical cross-linking agent to lower the cytotoxicity since EDC can easily be washed away as a water-soluble urea derivative [130]. Physical methods are not toxic to the cell environment but can cause denaturation of the protein. Examples of these methods are dehydrothermal (DHT) treatment and ultraviolet (UV) irradiation [90]. Since proteins are natural polymers with a natural structure, batch-to-batch consistency can also be a problem. Between batches, there can be differences in composition, amount of functionalized groups substituted, the secondary structure of the protein, water absorption, mechanical properties, and cell viability and biodegradability. These deviations give poor control over the ink [36,110]. When designing the ink, the protein’s synthesis route has to be chosen that offers reproducible and controllable results between batches. For commercial protein-based inks such as GelMA, (a gelatin-based ink), the maintenance of batch-to-batch consistency remains a problem [110]. Another concern that may limit the applicability of protein-based materials is the need for sterilization. This is because to bridge the gap between research successfully and applied in clinical practice, adequate biomaterial sterilization is needed.

Sterilization may result in unfavorable effects on the protein-based material. For instance, sterilization via heat treatment may affect the structural properties of biodegradable polymers such as protein-based materials [131]. Furthermore, although irradiation with gamma rays, electron beam or UV rays is done at low temperatures and leaves no residues, it may still induce changes in structural properties. Gamma radiation sterilizes by breaking down DNA, thus killing the pathogens. However, by breaking down the DNA, the radicals could cross-link polymers and change the properties of the protein-based biomaterial. It is important to note that during the irradiation, the temperature of the biomaterial increases to 30–40 °C. Electron beam sterilization works similarly to the radiation sterilization, but electron beam radiation is faster and less penetrative, thus, it is decreasing the possibility of biomaterial degradation. Nevertheless, during the irradiation, the temperature of the biomaterial might increase to about 50 °C for polymer biomaterials, enhancing the risk of alteration of the structure of the protein based material [132]. Plasma sterilization presents low temperature, improves cell interaction, increases wettability on surface of biodegradable polymers and is fast although it may also cause changes in chemical and mechanical properties of polymers and can leave reactive species [133]. Chemical treatments using chemicals such as ethanol, ethylene oxide and iodine present low temperatures and do not require complex equipment, nevertheless, they induce structural and biochemical property changes and leave toxic and carcinogenic residues. Ethylene oxide sterilization is the most popular chemical method because of its compatibility with many biomaterials. It is based on its strong alkylating property that disrupts cells and DNA, the clotting of proteins and the inactivation of enzymes. The processes, however, occur at the temperature range from 40 to 60 °C, and which is the range of temperature capable of degrading the protein-based biomaterial. The use of ozone and hydrogen peroxide in sterilization may lead to the oxidation of the protein and cell walls, leading to the destruction of the protein. It is therefore evident that no ideal sterilization technique can achieve exceptional sterilization for a wide variety of protein-based materials in the absence of post-sterilization effects. As a result, the operation conditions of a chosen sterilization technique should be accurately controlled and evaluated case by case.

## 7. Conclusions and Future Perspective

Advanced protein-based inks have enabled scientists to extend the range of protein application possibilities in tissue engineering via 3D printing technologies. These printing technologies explore the exceptional properties of proteins such as biocompatibility, cell attachment and differentiation in next-generation ink production. Some protein-based inks were also shown to be stimuli-responsive inks that enable the printed structures’ rapid response to local variations in body conditions. These variations can be induced by several externalities such as injuries and diseases using these protein-based stimuli-responsive constructs, enabling a targeted and a time variable response. The present review also highlighted the increasing interest in protein-based self-assembling inks due to their self-healing properties and their ability to form complex scaffolds due to self-induced cross-link formation. Protein-based inks also facilitate the introduction of new and improved properties, which can be incorporated into printing materials, thus promoting the growth of additive manufacturing in tissue engineering. Notably, the poor mechanical integrity of most protein-based inks was identified as constituting a possible challenge due to the soft nature of proteins.

Furthermore, since proteins are natural polymers, batch-to-batch consistency needs to be considered to gain enough control over the process. To resolve these issues the exploration of protein-based composite systems was discussed. It was also shown that protein-based inks outperform synthetic polymers with respect to biocompatibility, although there is a risk that the use of protein-based inks leads to an immune response. It is therefore important to explore approaches to reduce the immune response. We anticipate that the resolution of the issues and the sustained exploration of protein-based inks will enhance their state-of-the-art applications such as in the implementation of the scaffold-free printing and 3D organ-on-chip bioprinted systems.

## Figures and Tables

**Table 1:** Summary of advantages and disadvantages of some protein-based materials with regards to printing and applicability in tissue engineering.

Protein	Source	Advantages	Disadvantages	Some Remarks	Ref
Silk fibroin	Silkworm	High mechanical strength, extensibility, easy processability, high solubility in an aqueous medium, and printing fidelity. Scalable Young’s moduli (from 10 KPa and to 10 GPa) and wide distribution of pore size (0.5–100 µm). Furthermore, due to its amphiphilic nature, a precise volume of fibroin protein drops can be generated by optimizing the ink’s rheological properties at a wide range of pH values and ionic strengths.	Silk fibroin inks display shear-thinning behavior at low concentrations and are therefore not suitable for printing at low concentrations. It also shows Newtonian fluid behavior, thus creating difficulty when passing through the small nozzle diameter of the print head. The extrusion process is also usually interrupted due to clogging at the nozzle. The use of this protein may also lead to shear-induced conformational changes from random coil to β-sheet and crystallite formation. Fibroin scaffolds might biodegrade very slowly and present weak cell affinity.	When modified with methacrylate groups, silk-based biomaterial ink can be printed using digital light processing to yield highly complex structures with structural stability and reliable biocompatibility.	[6,36,37,38,39]
	Spider	High mechanical strength (dragline), extensibility and good shape fidelity. It promotes good cell adherence, cell viability and proliferation. It presents shear thinning behavior and can be printed without cross-linkers or additives to enhance mechanical stability. Due to physical cross-links and reversible gelation upon shear thinning, the biomaterial ink does not clog at the nozzle.	It is relatively difficult to achieve the same quality during mass production of this protein, making it less suitable for biofabrication purposes.	Development of recombinant proteins must be developed Cell viability is lower compared to other inks such as gelatin.	[28,40,41]
Keratin	Sheep wool	It is characterized by a high fracture strength (180–260 MPa).	Keratin has low extensibility. The mechanical properties also vary with air relative humidity.		[25]
	Human hair	Scaffolds with compression modulus ranging from 5.49 to 15.45 kPa and open pores with a diameter ranging from 10 to 30 μm.		The 3D keratin scaffolds were produced via UV crosslinking activated by a riboflavin-persulfate-hydroquinone solution.	[42]
Gelatin	Gelatin	It favors cell proliferation. The methacrylated gelatin has been widely used to develop photo-crosslinked hydrogels (especially in 3D printing). It may also be used to fabricate skin substitutes and has the capacity for gel suspension at low temperatures. It has proper viscosity for printing. There are domains for cell-adhesives. Scaffolds might be printed with pore sizes ranging from 200 to 600 µm, but the differentiation and infiltration of mesenchymal stromal cells seem to be more favored in pores larger than 500 µm. Crosslinking gelatin with methylenebisacrylamide might lead to scaffolds with a compressive modulus of 20 kPa, mimicking the adipose tissue.	The extent of biocompatibility of gelatin may depend on the source of gelatin. The protein, however, has poor mechanical properties. There is also a risk of degradation at temperature greater than 37 °C.	Better cytocompatibility than keratin. It may also dissolve in water at temperatures above 30 °C. Notably, gelatin may be incorporated in silk scaffold fabrication to reduce the risk of degradation.	[43,44]
Collagen	Porcine	It is characterized by high porosity, tensile strength and biodegradability. 3D printed collagen scaffolds presented a porosity of 90%. This protein is abundant and has cross-linking capacity. It can also form gels as the temperature changes. There are cell adhesive domains in collagen structure. Cell viability in printed collagen is good under optimized values of printing parameters and cell densities.	There is a possibility for lack of biocompatibility and the risk of batch-to-batch variations. Has poor mechanical properties and low stiffness. Immunogenicity concern limits its applications. Gelation time is long. Specifically, fish collagen has a low denaturation temperature which might limit its printing capacity.	Collagen exhibits superior cytocompatibility compared to keratin and gelatin.	
	Fish				[13,35,36]

**Table 2:** Comparison of biofabrication techniques used for protein-based inks [39,46,48].

Biofabrication Technique	Protein-Based Ink Compatibility	Advantages	Disadvantages
Inkjet printing	Ideally used with low-viscosity inks thus it can be applied for use on some of the protein-based inks due to their ability of self-assembly after printing.	It can achieve a high resolution when employed in fabricating proteins. It is a low-cost and high-speed approach that facilitates the maintenance of high cell viability.	The use of this approach may cause the constructed structure to have poor structural integrity. Furthermore, high viscous inks are limited with low precision in droplet size and positioning is also a challenge. This technique may be difficult to be employed when using protein-based inks since proteins are viscous biopolymers and they do not exhibit shear thinning behavior at concentrations > 20 wt %.
Extrusion printing	Suitable for protein-based inks since it is applicable in a wide viscosity range.	It facilitates the bioprinting of inks with high cell density.	This approach presents limited resolution. It may also reduce the viability of cells and is characterized by low speed.
Laser-based printing	Suitable for protein-based inks since it is applicable in a wide viscosity range.	Facilitates the bioprinting of inks with high cell density.	This approach is costly and time-consuming. The use of this technique may lead to the generation of heat that may affect cells.
Stereolithography	This technique is suitable for photosensitive protein-based inks.	This technique can achieve a high resolution and accuracy.	This approach is costly and is only applicable to photosensitive protein-based inks.
Electrospinning	The suitability of this technique depends on the viscosity of the protein-based ink.	This technique produces very thin fibers characterized by enhanced mechanical properties—relatively low cost.	The electrospinning technique can only enable limited scaffold volume. There are also risks that the solvent used may be toxic.
Melt electrospinning	The suitability of this technique depends on the viscosity of the protein-based ink.	This technique does not require solvent-free and is recognised as environment-friendly. The technique enables better control over fiber deposition.	There may be some limitations when using proteins due to its wide range of viscosities. More tests are therefore required to assess the biomaterials.

**Table 3:** Some protein-based materials that are employed in 3D printing.

Protein-Based Material	Printing Method	Remarks	Ref
Silk fibroin/glycidyl methacrylate	Digital light processing	Good cell proliferation	[77]
Gelatin/polyethylene glycol cross-linkers	Extrusion	Neonatal fibroblast viability was supported, promoted cell proliferation	[78]
Collagen I/riboflavin	Stereolithography	The resulting constructs were shown to have excellent mechanical properties and support cell proliferation.	[79]
Collagen/chitosan/α, β-glycerophosphate	Not reported	The cell viability was reported to vary with biomaterials proportion	[61]
Methacryloyl-recombinant-tropoelastin based	2-photon polymerization	The modification of proteins could lead to the formation of both methacrylamide and methacrylate groups	[80]
Collagen/ECM-alginate	Extrusion	Osteogenic activities in the composite bioink containing collagen were shown to be improved compared to an only alginatebased bioink	[81]
Amphiphile peptides and keratin–ECM proteins	Droplet-on-demand inkjet	Using a 500 μm nozzle diameter, cell viability of >88% was maintained	[63]
Alginate–PLA nanofibers	Extrusion	Higher levels of cell proliferation were reported within bioprinted strands	[82]
Gelatin–alginate–carbon nanotubes	Extrusion	Cell proliferation was supported with proper doping of carbon nanotubes shown to increase the mechanical properties of the composite scaffolds	[83]

**Table 4:** Protein-based 3D printed materials in brain tissue applications.

Printing Technique	Type of Protein	Gelation Method	Inner Diameter (mm)	Cell Concentration (million/mL)	Printing Pressure (kPa)	Cell Type	Cell Viability	Ref
Melt Electrowriting	Matrigel (reinforced with PCL)	_	_	_	300	Cortical neurons	1 day = 85 ± 7% 7 days = 83 ± 6% 14 days = 65 ± 7% 21 days = 54 ± 8%	[101]
Microfluidic (Lab-On-The-Printer)	Fibrin (+alginate)	Chemical cross-linking—CaCl_2_ (+thrombin and chitosan + genipin to avoid chitosan cross-linking)	_	1	Ink = 5 Cross-linker = 6Buffer channel = 10	hiPSCs-derived NPCs	1 day = 90% 7 days = 95%	[102]
Microfluidic (Lab-On-The-Printer)	Fibrin (+alginate and genipin)	Chemical cross-linking with CaCl_2_ (+chitosan and thrombin)	_	_	_	GBM (glioblastoma multiforme) cells	After printing = 88.78 ± 2.92% 3 days = 98.09 ± 0.89% 6 days = 91.78 ± 5.96% 9 days = 83.93 ± 5.75% 12 days = 86.12 ± 5.09%	[103]
DLP 3D printing	Gelatin (GelMA)	Photocross-linking	1.2, 1.6, 2.0	-	_	PC-12 (rat’s pheochromocytoma)	1 day = 97.2%, 95.6%, 35.1%	[104]
Extrusion (3D Bio-Plotter)	Fibrin + RGD-peptide (+alginate and hyaluronic acid)	Chemical cross-linking with CaCl_2_ + thrombin	_	1	_	Schwan cells (isolated from sciatic nerve)	1 day = 89% 10 days = 95%	[105]
Extrusion + freeze drying	Collagen and silk fibroin	Collagen gel formed via dialysis with deionized water at 4 °C	0.210	20	_	NSCs (neural stem cells)	_	[65]
Organ-on-Chip	Matrigel	Thermal cross-linking at 37 °C in 5% CO_2_ atmosphere	_	_	_	hiPSCs-derived GABAergic neurons (+astroctyes)	_ (Assessed after exposure the toxic agent)	[109]
In-house built printer	Collagen type I (+PPy-b-PCL)	Thermal cross-linking at 4 °C (fridge)	0.5	0.1	_	PC-12 cells (rat’s pheochromocytoma)	_ (Assessed with absorbance test after 2 days to check the difference between pure collagen and collagen + PPy − b − PCL hybrid)	[108]

**Table 5:** Commercially available 3D printable protein/polypeptide-based hydrogels [111,112,113,114,115,116,117].

Company Name	Product Name	Hydrogel Type, Composition, and Properties	Application Notes and Properties
BIOGELX	BiogelxTM-INK-S	A synthetic peptide hydrogel ink	This ink presents gelation that is independent of variations in temperature and pH values.
	BiogelxTM-INK-Arg-Gly-Asp (RGD)	A fibronectin-functionalized synthetic peptide hydrogel ink	Gelation is independent of variations in temperature and pH values. This ink also employs a tripeptide of arginine, glycine, and aspartate as a surface ligand for enhanced functionality.
	BiogelxTM-INK-GFOGER	Collagen-functionalized synthetic peptide	Gelation is independent of temperature and pH. To enable enhanced functionality, the hexapeptide of GFOGER is employed as a surface ligand for enhanced functionality.
Manchester BIOGEL	Standard or functional PeptiInks®	Neutral or charged. Fibronectin, laminin or collagen Alpha 1, G′ (kPa) = 5 Alpha 2, G′ (kPa) = 10 alpha 4, G′ (kPa) = 1 functionalized with = RGD, IKVAV, YIGSR, GOFGER	Due to the shear thinning characteristics, it can be employed in the extrusion-based printer. Additionally, encapsulation of cells is possible, and thus, the cells may have enhanced structural stability and long-term viability when printed directly.
Regmat-3d	Fibronectin	Functionalized synthetic peptide hydrogel ink	This ink is capable of mimicking the extracellular matrix via the formation of a nanofibrous network. It is biocompatible and can be utilized in different printing applications since its mechanical and chemical properties can be modified.
Gelomics	Gelatin Methacryloyl (GelMA)—Porcine	It is based on porcine gelatin (type A). It is also characterized by a degree of methacrylation ranging from 75% to 85%	This ink can be reconstituted in phosphate buffered saline or (4-(2-hydroxyethyl)-1-piperazineethanesulfonic acid) buffer while maintaining the desired concentration. The ink can also be combined with a photoinitiator, thus making the resulting hydrols photocross-linkable. Stability at body temperature is also achieved.
	Gelatin Methacryloyl (GelMA—Bovine)	It is based on bovine gelatin (type B). It is also characterized by a degree of methacrylation ranging from 75% to 85%	Similar to the gelatin methacryloyl (GelMA)–porcine, this ink can also be reconstituted in phosphate buffered saline or (4-(2-hydroxyethyl)-1-piperazineethanesulfonic acid) buffer while maintaining the desired concentration. The ink can also be combined with a photoinitiator, thus making the resulting hydrols photocross-linkable. Stability at body temperature is also achieved.
	LunaGel™	It is characterized by a photocross-linkable extracellular matrix that is based on either the bovine bone or porcine skin gelatin. This ink is composed of collagens of type I, III, IV, and V. It also contains connective tissue glycoproteins and proteoglycans	The ink may exist as a low stiffness (0–6.5 kPa) or a high stiffness kit (0–25 kPa).
Brinter-bio inks	Fibrinogen, collagen I and gelatin	-	-
Advanced Biomatrix	Lifeink® 200	The ink has a pH value of 7, and is isotonic, indicating its readiness for cell addition and printing.	It is essentially a neutralized type I bovine collagen ink
	Lifeink® 240	This in has a pH value of <7 (i.e., acidic) and it is categorized as a type I collagen based ink	It can be used to yield high resolution collagen scaffold, after neurtalization.
Corning	Corning® PuraMatrix™	Collagen I, bovine or rat tail tendon or human placenta, collagen III, human placenta, collagen IV, laminin, mouse, fibronectin, human, collagen VI	It is a synthetic matrix that can be used to create 3D microenvironments for various cell culture experiments. The matrix is capable of self-assembly, under physiological conditions, via the peptide component’s self-assembly into a 3D hydrogel. A fibrous structure on a nanometer-scale characterizes the resulting 3D hydrogel.
Cellink	Cellink fibrin	Contains fibrinogen	This ink is capable of developing a stable compound network using thrombin and an ionic binding agent. Cellink can also provide a physiologically relevant wound-healing environment after cross-linking. Cellink fibrin also contains in situ fibrin and fibrinogen after cross-linking.
	GelXA ink	Contains fibrinogen	The presence of dual-cross-linking capabilities characterizes GelXA; the dual-cross-linking capabilities are achieved via photocuring and treatment with an ionic thrombin-containing cross-linking agent.
	Cellink gelma	GelMA A: GelMA and alginate	This ink has shear-thinning rheological properties thus can be printed at low pressures for filament formation once deposited.
		GelMA HA:	This ink is composed of GelMA base, xanthan gum and alginate and is characterized by enhanced printability, ease of use, and stability. The ink can also be used to facilitate photoinitiator-assisted cross-linking, ionic cross-linking, or a combination of both.
		GelMA HA:	This ink is composed of GelMA base and xanthan gum
		GelMA C: GelMA and nanofibrillated cellulose	This ink is composed of GelMA and nanofibrillated cellulose. It is characterized by smooth printability at room temperature without temperature control. The ink also provides fibrillar morphology for the benefit of specific cell. The ink is also capable of rapid cross-linking via photocuring in the absence of an ionic cross-linking solution.
Cellink laminink	111, 121, 411, 521, and LAMININK+	This ink is composed of three subunits—*α*-chain, *β*-chain and *γ*-chain. The different inks may be used for different specialized cells such as hepatocytes, cardiomyocytes neurons etc.	
Bio conductink	Gleatin gelatin methacrylate ink with 0.25 percent of photoinitiator (LAP)	This ink can conduct electrical charges and may be used for muscular contraction and neural tissue models. The ink also presents temperature sensitive printability	

## Data Availability

Not Applicable.
